# A Comparison of RBF Neural Network Training Algorithms for Inertial Sensor Based Terrain Classification

**DOI:** 10.3390/s90806312

**Published:** 2009-08-12

**Authors:** Tuba Kurban, Erkan Beşdok

**Affiliations:** Geomatics Engineering, Engineering Faculty, Erciyes University, Turkey E-Mail: tubac@erciyes.edu.tr (T.K.)

**Keywords:** radial basis neural networks, terrain classification, inertial navigation sensors, genetic algorithm, artificial bee colony algorithm, Kalman filtering, gradient descent

## Abstract

This paper introduces a comparison of training algorithms of radial basis function (RBF) neural networks for classification purposes. RBF networks provide effective solutions in many science and engineering fields. They are especially popular in the pattern classification and signal processing areas. Several algorithms have been proposed for training RBF networks. The Artificial Bee Colony (ABC) algorithm is a new, very simple and robust population based optimization algorithm that is inspired by the intelligent behavior of honey bee swarms. The training performance of the ABC algorithm is compared with the Genetic algorithm, Kalman filtering algorithm and gradient descent algorithm. In the experiments, not only well known classification problems from the UCI repository such as the Iris, Wine and Glass datasets have been used, but also an experimental setup is designed and inertial sensor based terrain classification for autonomous ground vehicles was also achieved. Experimental results show that the use of the ABC algorithm results in better learning than those of others.

## 1. Introduction

Agricultural applications, search/rescue missions, surveillance, supply and logistics are some of the operational fields of autonomous ground vehicles. These operations may necessitate for traversing some off-road or indoor terrains that can have an effect on the vehicle performance [1]. In such operations, determining the environment of the vehicle improves its efficiency. Estimation of the current terrain type can be useful since the terrain conditions affect both planning and motion control stages [2]. An automated terrain-dependent driving process can be obtained with terrain classification. In the literature, computer vision based algorithms are commonly used to classify the terrain which is being traversed [3,4]. However, these methods can be unreliable and their performance mostly depend on the lighting conditions. Vibrations of the ground vehicles obtained by inertial sensors can also be used to classify or characterize the terrain [5].

A radial basis function (RBF) network is a special type of neural network that uses a radial basis function as its activation function [6]. RBF networks are very popular for function approximation, curve fitting, time series prediction, control and *classification* problems. The radial basis function network is different from other neural networks, possessing several distinctive features. Because of their universal approximation, more compact topology and faster learning speed, RBF networks have attracted considerable attention and they have been widely applied in many science and engineering fields [7–11].

In RBF networks, determination of the number of neurons in the hidden layer is very important because it affects the network complexity and the generalizing capability of the network. If the number of the neurons in the hidden layer is insufficient, the RBF network cannot learn the data adequately; on the other hand, if the neuron number is too high, poor generalization or an overlearning situation may occur [12]. The position of the centers in the hidden layer also affects the network performance considerably [13], so determination of the optimal locations of centers is an important task. In the hidden layer, each neuron has an activation function. The gaussian function, which has a spread parameter that controls the behavior of the function, is the most preferred activation function. The training procedure of RBF networks also includes the optimization of spread parameters of each neuron. Afterwards, the weights between the hidden layer and the output layer must be selected appropriately. Finally, the bias values which are added with each output are determined in the RBF network training procedure.

In the literature, various algorithms are proposed for training RBF networks, such as the gradient descent (GD) algorithm [14] and Kalman filtering (KF) [13]. These two algorithms are derivative based and have some weaknesses such as converging to a local minima and time-consuming process of finding the optimal gradient. Because of these limitations, several global optimization methods have been used for training RBF networks for different science and engineering problems such as genetic algorithms (GA) [15], the particle swarm optimization (PSO) algorithm [12], the artificial immune system (AIS) algorithm [16] and the differential evolution (DE) algorithm [17].

The ABC algorithm is a population based evolutional optimization algorithm that can be applied to various types of problems. The ABC algorithm is used for training feed forward multi-layer perceptron neural networks by using test problems such as XOR, 3-bit parity and 4-bit encoder/decoder problems [18]. In this study, training the RBF network with ABC algorithm has been proposed and the training performance of the ABC algorithm has been compared with GD [14], KF [13] and GA on classification test problems and an inertial sensor based terrain classification problem.

The rest of the paper is organized as follows: Section 2 presents *Radial Basis Function Networks* and Section 3 gives brief descriptions of training algorithms of RBF networks. Section 4 illustrates the *Artificial Bee Colony (ABC) Algorithm*. Section 5 presents *Training of RBF Neural Networks by Using ABC*. In Section 6, the performance of training algorithms of RBF network that is applied to classification problems have been evaluated and experimental results have been given. Finally, in the last section, some concluding remarks are presented.

## 2. Radial Basis Function Networks

**Figure 1.**
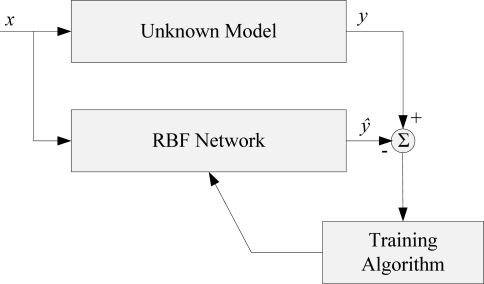
Block diagram of a RBF network.

Neural networks are non-linear statistical data modeling tools and can be used to model complex relationships between inputs and outputs or to find patterns in a dataset. RBF network is a type of feed forward neural network composed of three layers, namely the input layer, the hidden layer and the output layer. Each of these layers has different tasks [17]. A general block diagram of an RBF network is illustrated in Figure 1.

In RBF networks, the outputs of the input layer are determined by calculating the distance between the network inputs and hidden layer centers. The second layer is the linear hidden layer and outputs of this layer are weighted forms of the input layer outputs. Each neuron of the hidden layer has a parameter vector called center. Therefore, a general expression of the network can be given as [19]:

(1) $${\hat{y}}_{j}=\sum_{i=1}^{I}w_{\mathrm{ij}}\;\phi \left(\left\|x-c_{i}\right\|\right)+\beta_{j}$$

The norm is usually taken to be the Euclidean distance and the radial basis function is also taken to be Gaussian function and defined as follows:

(2) $$\varphi (r)=\text{exp}\left(-\alpha_{i}\cdot {\left\|x-c_{i}\right\|}^{2}\right)$$

where,

**Table:** 

*I*	Number of neurons in the hidden layer	*i* ∈ {1,2,…,*I*}
*J*	Number of neurons in the output layer	*j* ∈ {1,2,…,*J*}
*w_ij_*	Weight of the *i*^th^ neuron and *j*^th^ output	
*ϕ*	Radial basis function	
*α_i_*	Spread parameter of the *i*^th^ neuron	
**x**	Input data vector	
**c***_i_*	Center vector of the *i*^th^ neuron	
*β_j_*	Bias value of the output *j*^th^ neuron	
*ŷ_j_*	Network output of the *j*^th^ neuron	

**Figure 2.**
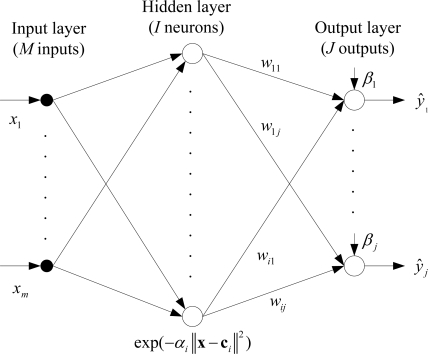
Network architecture of the RBF.

Figure 2 shows the detailed architecture of an RBF network. *M* dimensional inputs (*x_1_*,…,*x_m_*) are located in the input layer, which broadcast the inputs to the hidden layer. The hidden layer includes *I* neurons and each neuron in this layer calculates the Euclidean distance between the centers and the inputs. A neuron in the hidden layer has an activation function called the basis function. In the literature, the Gaussian function is frequently chosen as the radial basis function and it has a spread parameter to shape the curve (*α_1_*,…,*α_i_*). The weighted (*w_11_*,…,*w_ij_*) outputs of the hidden layer are transmitted to the output layer. Here, *I* (*i* ∈ {1,2,…,*I*}) denotes the number of neurons in the hidden layer and *J* (*j* ∈ {1,2,…,*J*}) denotes the dimension of the output. The output layer calculates the linear combination of hidden layer outputs and bias parameters (*β_1_*,…,*β_j_*). Finally, the outputs of the RBF network are obtained (*ŷ_1_*,…,*ŷ_j_*).

The design procedure of the RBF neural network includes determining the number of neurons in the hidden layer. Then, in order to obtain the desired output of the RBF neural network *w*, *α*, c and *β* parameters might be adjusted properly. Reference based error metrics such as mean square error (MSE) or sum square error (SSE) can be used to evaluate the performance of the network. Error expression for the RBF network can be defined as follows:

(3) $$E^{\text{SSE}}\;(w,a,c,\beta )=\sum_{j=1}^{J}{\left(y_{j}-{\hat{y}}_{j}\right)}^{2}$$

Here *y_j_* indicates the desired output and *ŷ_j_* indicates the RBF neural network output. The training procedure of the RBF neural network involves minimizing the error function.

## 3. Training Algorithms of RBF Networks

This section gives brief descriptions of training algorithms of RBF networks which were used in this paper for comparison purposes. The artificial bee colony (ABC) algorithm, which is newly applied to RBF training, is explained in detail in Section 4.

### 3.1. Gradient Descent (GD) Algorithm

GD is a first-order derivative based optimization algorithm used for finding a local minimum of a function. The algorithm takes steps proportional to the negative of the gradient of the function at the current point. In [13], the output of a RBF network has been written as:

(4) $$\hat{y}=\left[\begin{array}{ccccc} w_{11} & w_{11} & . & . & w_{1J}\\ w_{21} & w_{21} & . & . & w_{2J}\\ . & . & . & . & .\\ . & . & . & . & .\\ w_{I1} & . & . & . & w_{\mathrm{IJ}} \end{array}\right]\cdot \left[\begin{array}{c} 1\\ \varphi \left({\left\|x-c_{1}\right\|}^{2}\right)\\ .\\ .\\ \varphi ({\left\|x-c_{I}\right\|}^{2} \end{array}\right]$$

and

(5) $$\hat{Y}=W\cdot H$$

where the weight matrix is represented as *W* and *φ* matrix is represented as *H*. GD algorithm can be implemented to minimize the error after defining the error function:

(6) $$E=\sum {\left(Y-\hat{Y}\right)}^{2}$$

where *Y* is the desired output. RBF can be optimized with adjusting the weights and center vectors by iteratively computing the partials and performing the following updates:

(7) $$\begin{array}{l} w_{\mathrm{ij}}=w_{\mathrm{ij}}-\eta \frac{\partial E}{\partial w_{\mathrm{ij}}}\;\\ c_{i}=c_{i}-\eta \frac{\partial E}{{\partial c}_{i}} \end{array}$$

where *η* is the step size [13].

### 3.2. Kalman Filtering (KF) Algorithm

The state of a linear dynamic system can be efficiently estimated by Kalman filter from a series of noisy measurements. It is used in a wide range of engineering applications from INS/GPS integration [20] to computer vision and many applications in control theory. A nonlinear finite dimensional discrete time system has been considered in [13] as:

(8) $$\begin{array}{l} \theta_{k+1}=\theta_{k}+\omega_{k}\\ y_{k}=h\left(\theta_{k}\right)+v_{k} \end{array}$$

where the vector *θ_k_* is the state of the system at time *k*, *ω_k_* is the process noise, *y_k_* the observation vector, *v_k_* is the observation noise and *h* is the nonlinear vector function of the state.

KF can be used to optimize weight matrix and center vectors of RBF as a least squares minimization problem. In a RBF network, *y* denotes the target output vector and *h*(*θ_k_*) denotes actual output of the *k*th iteration of the optimization algorithm.

(9) $$\begin{array}{l} y={\left[y_{1}\;\;\ldots \;\;y_{J}\right]}^{T}\\ h\left(\theta_{k}\right)={\left[{\hat{y}}_{1}\;\;\ldots \;\;{\hat{y}}_{J}\right]}^{T} \end{array}$$

The vector *θ* thus consists of all RBF parameters:

(10) $$\theta ={\left[w_{11}\;\;\cdots \;\;w_{\mathrm{IJ}}\;\;\;c_{1}\;\;\cdots \;\;c_{I}\right]}^{T}$$

For a detailed explanation of the use of GD and KF for RBF training see [13].

### 3.3. Genetic Algorithms (GA)

GA is an optimization method used in many research areas to find exact or approximate solutions to optimization and search problems. Inheritance, mutation, selection and crossover are the main aspects of GA that inspired from evolutionary biology. The population refers to the candidate solutions. The evolution starts from a randomly generated population. For all generations, the fitness (typically a cost function) of every individual in the population is evaluated and the genetic operators are implemented to obtain a new population. In the next iteration the new population is then used. Frequently, GA terminates when either a maximum number of generations has been reached, or a predefined fitness value has been achieved. In RBF training, the individuals consist of the RBF parameters such as weights, spread parameters, center vectors and bias parameters. The fitness value of an individual can be evaluated using an error function such as MSE or SSE of the desired output and the actual output of the network.

## 4. The Artificial Bee Colony (ABC) Algorithm

**Figure 3.**
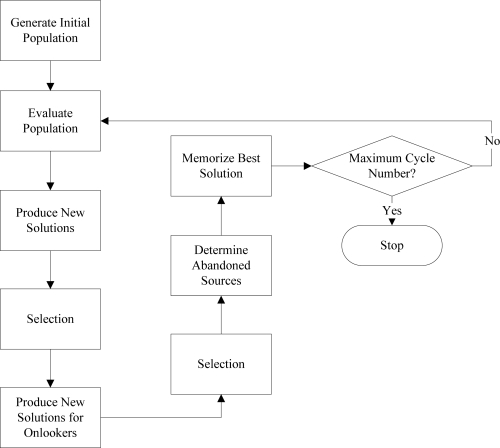
Basic flowchart of the ABC algorithm.

The Artificial Bee Colony algorithm is a heuristic optimization algorithm proposed by Karaboga in 2005 [21]. The ABC algorithm has been inspired by honey bees’ intelligent foraging behavior. In the ABC model, the colony consists of three different bee groups, namely worker bees, onlooker bees and scout bees. For each food source there is only one associated employed bee. So, the number of the worker bees indicates the number of the food sources. Honey bees’ intelligent foraging behavior can be explained as follows: worker bees go to their food sources, after determining the nectar amount for that food source they explore new neighboring food sources. Then, they come back and dance around the hive. Onlooker bees which are watching the dance of the worker bees, choose a food source according to the worker bees’ dances. Probability of the food source that will be chosen is related with the quality of the food nectar and the left food amount. If a food source cannot be improved further through a predefined number of cycles, then the source is abandoned. Subsequently, randomly produced new sources are replaced with the abandoned ones by scouts. The best food source is determined and position of that food source is memorized. This cycle is repeated until requirements are met [21–23]. The basic flowchart of the ABC algorithm is illustrated in Figure 3.

In the ABC algorithm, a food source indicates a possible solution of the optimization problem and the nectar amount of the food source indicates the fitness value of that food source. The number of worker bees corresponds to the possible solutions. First, an initial randomly distributed population is generated. After initialization, a search cycle of the worker, onlooker and scout bees in the population is repeated, respectively. A worker bee changes the food source and discovers a new food source. If the nectar amount of the new source is more than the old one, the worker bee learns the new position instead of the old one. Otherwise it keeps the old position. After all the worker bees complete the search process; they share the position information with the onlooker bees. Onlooker bees evaluate the nectar amounts and choose a food source. The probability value of a food source is calculated by using:

(11) $$p_{i}=\frac{f_{i}}{\sum_{n=1}^{\mathrm{SN}}f_{n}}$$

where *p_i_* is the probability value of the source *i*, *f_i_* is the fitness value of the solution *i* that is proportional with the nectar amount and *SN* is the number of the food sources or worker bees.

ABC algorithm uses equation 12 to obtain a new food position from the old position in the memory:

(12) $$v_{\mathrm{ij}}=x_{\mathrm{ij}}+\Theta_{\mathrm{ij}}\;\left(x_{\mathrm{ij}}-x_{\mathrm{kj}}\right)$$

where *j* ∈ {1,2,…,*SN*} and *i* ∈ {1,2,…,*D*} are the randomly selected indices and *j* must be different from *i. D* indicates the number of the parameters to be optimized. Θ*_ij_* is a random number between [−1, 1] and this number controls the production of the food sources around x_ij_ As can be seen from equation 12, if the difference between x_ij_ and x_kj_ decreases, the step size get a decrease, accordingly. Therefore, the step size is adaptively modified while the algorithm reaches the optimal solution in the search area. The food source which does not progress for a certain number of cycles is abandoned. This cycle number is very important for ABC algorithm and is called “limit”. Control parameters of the ABC algorithm are number of the source (*SN*), limit parameter and number of the maximum cycle [24,25].

## 5. Training of RBF Neural Networks by Using ABC

As mentioned before, training of an RBF neural network can be obtained with the selection of the optimal values for the following parameters:

- weights between the hidden layer and the output layer (*w*)
- spread parameters of the hidden layer base function (*α*)
- center vectors of the hidden layer (*c*)
- bias parameters of the neurons of the output layer (*β*)

The number of neurons in the hidden layer is very important in neural networks. Using more neurons than that is needed causes an overlearned network and moreover, increases the complexity. Therefore, it has to be investigated how the numbers of neurons affect the network’s performance.

The individuals of the population of ABC include the parameters of the weight (*w⃗*), spread (*α⃗*), center (*c⃗*) and bias (*β⃗*) vectors. An individual of the population of ABC algorithm can be expressed as:

(13) $$P_{i}=\left[\vec{w}\;\;\vec{\alpha }\;\;\vec{c}\;\;\vec{\beta }\right]$$

The quality of the individuals (possible solutions) can be calculated using an appropriate cost function. In the implementation, SSE between the actual output of the RBF network and the desired output is adopted as the fitness function:

(14) $$f=E^{\text{SSE}}$$

## 6. Experiments

The experiments conducted in this study are divided into two sub-sections. First, comparisons on well-known classification problems obtained from UCI repository are given. The second part of this section deals with terrain classification problem using inertial sensors in which the data was obtained experimentally.

### 6.1. Comparison of Algorithms on Test Problems

In the experiments of test problems, the performance of RBF network trained by using ABC is compared with GA, KF and GD algorithms. The well-known classification problems—Iris, Wine and Glass—which were obtained from UCI repository [26] are used.

For all datasets, experiments are repeated 30 times. For each run, datasets are randomly divided into train and test subsets. 60% of the data set is randomly selected as the training data and remained data set is selected as the testing data. Afterwards, average and standard deviation of the 30 independent runs are calculated. General characteristics of the test and train dataset are illustrated in Table 1.

One of the most important design issue of an RBF network is the number of the neurons in the hidden layer. Therefore, the experiments are conducted on different RBF networks which has 1 neuron to 8 neurons located in the hidden layer.

In the experiments, learning parameter of GD is selected as *η* = 0.01 [13]. For the KF algorithm *P0* = 40*I*, *Q* = 40*I*, and *R* = 40*I* are chosen [13]. Here, *I* denotes the appropriate sized identity matrix. The control parameters of GA and ABC used in the experiments are given at Tables 2 and 3, respectively.

In the experiments percent of correctly classified samples (PCCS) metric is used as the performance measure:

(15) $$\mathrm{PCCS}=\frac{\mathrm{Correctly}\;\mathrm{Classified}\;\mathrm{Samples}}{\mathrm{Total}\;\;\mathrm{Samples}}\times 100$$

The statistical results of 30 replicates are given at Tables 4—6 for Iris, Wine and Glass datasets, respectively. In the tables, the average PCCS values and the standard deviations (given in the parenthesis) are illustrated.

Gradient descent algorithm is a traditional derivative based method that is used for training RBF networks [14]. However, it has some drawbacks such as trapping at local minima and computational complexity in consequence of slow convergence rate.

Kalman filtering is another derivative based method and several studies are performed for training RBF network with Kalman filtering [13]. It converges in a few iterations so it reduces the computational complexity. Therefore, Kalman filtering is preferable over GD because of this feature.

GA is population based evolutional optimization algorithm and it has been used for training RBF network in several studies [27]. Because of non-derivative based characteristic, it is distinguished from previous two algorithms and it is more robust for finding the global minimum. However, population based methods have a disadvantage such as slow converging rate. In this study, MATLAB Genetic Algorithms Toolbox^®^ is used to perform the experiments with GA. Population size and generation/cycle count are the same with ABC settings for comparing the training performances.

ABC is an evolutional optimization algorithm that is inspired by the foraging behavior of honey bees. It is a very simple and robust optimization technique. The results show that the ABC algorithm is more robust than GA because of the changes in standard deviations. Average results of the training results show that the ABC algorithm is better than the other methods.

As can be seen from Tables 4—6, the performance of the ABC algorithm is better than the performances of the GD and KF methods. The performance of the ABC algorithm is nearly same with GA. However, standard deviations of the algorithms have pointed that ABC is more robust and stable than other methods. For the Iris, Wine and Glass datasets, standard deviations of the four algorithms can be seen in the tables. These results show that randomly selected data do not affect the performance of the ABC algorithm.

In addition, the number of neurons affects the network performance. As can be seen from the figures, as the number of neurons increases, the performance of the network does not increase accordingly. However, in the experiments it is realized that using three neurons gives acceptable results for the ABC algorithm. Other algorithms can reach the same performance by using more than three neurons. Since the number of neurons directly influences the time complexity of the algorithm, the required minimum number of neurons has to be used in the applications. In this context, the ABC algorithm is better than the others.

### 6.2. Comparison of Algorithms on Inertial Sensor based Terrain Classification

In this section, a terrain classification experiment is presented. The goal of this experiment is to identify the type of the terrain being traversed, from among a list of candidate terrains. Our proposed terrain classification system uses typically available an inertial measurement unit (IMU): XSens MTi-9 [28,29]. The Xsens MTi-9 sensor is a miniaturized, MEMS gyro-based Attitude and Heading Reference System whose internal signal processor provides drift-error free 3D acceleration, 3D orientation, and 3D earth-magnetic field data. The drift-error growing nature of inertial systems limits the accuracy of inertial measurement devices. Inertial sensors can supply reliable measurements only for small time intervals. The inertial sensors have been used in some recent research for stabilization and control of digital cameras, calibration patterns and other equipment [30].

**Figure 4.**
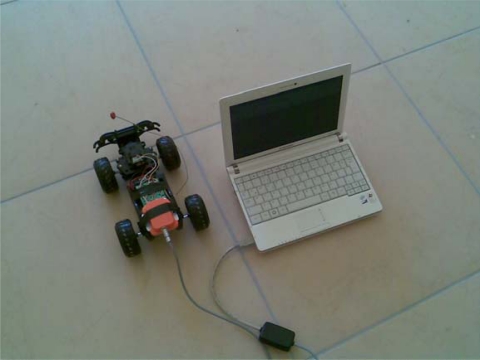
Experimental setup.

The experimental platform is shown in Figure 4. The Xsens IMU is attached to the body of the mobile vehicle and connected to the notebook PC with USB cable.

**Figure 5.**
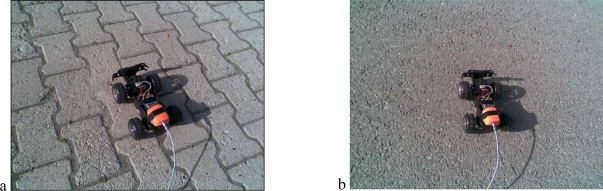

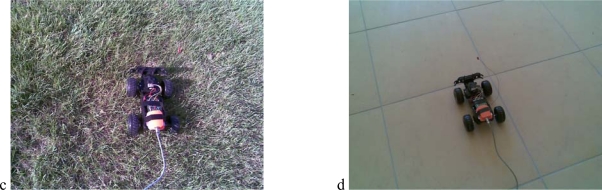
Terrain types: (a) pavement, (b) asphalt, (c) grass and (d) tile.

In this study, we try to identify which one of the four different candidate terrains the vehicle travelled on: pavement, asphalt, grass and tile. Our hypothesis is that the vibrations of different terrains influence the output of the IMU sensor. The data sampled at 100 Hz for 80 seconds duration for each terrain type. Afterwards, the data is preprocessed before classification using proposed RBF scheme. Outdoor terrain types analyzed in this study are pavement, asphalt and grass. For indoor applications tile floor is used. The terrain types are shown in Figure 5.

Previous experiments have shown that RBF networks are efficient classifiers. In this experiment the proposed RBF scheme is also used for terrain classification. RBF network structure for this problem has four outputs that help to identify the terrain type. Each output can vary from zero to one, in proportion to the likelihood that a given signal presented in the input of the RBF network belongs to one of the four subject terrains: pavement, asphalt, grass or tile. The RBF has *n* inputs corresponds to sensor data.

The *i*th sample of Xsens IMU can be given as:

(16) $$S_{i}=\left[{\mathrm{acc}}_{x}\;{\mathrm{acc}}_{y}\;{\mathrm{acc}}_{z}\;{\mathrm{gyr}}_{x}\;\;{\mathrm{gyr}}_{y}\;\;{\mathrm{gyr}}_{z}\;{\mathrm{mag}}_{x}\;{\mathrm{mag}}_{y}\;{\mathrm{mag}}_{z}\right]$$

where *acc* is 3D acceleration (m/s^2^), *gry* is 3D rate of turn (deg/s) and *mag* is 3D earth magnetic field (mGauss).

**Figure 6.**
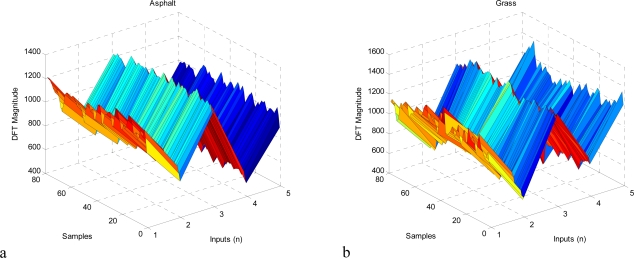

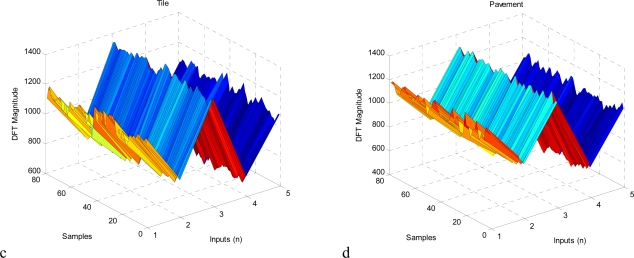
Preprocessed input data for terrain classification.

Discrete Fourier transform (DFT) is performed on the inertial data obtained from Xsens IMU. The sensor acquires data at 100 Hz. Therefore, the input signal at fixed intervals of 100 samples (1 second duration) is obtained and then DFT of the signal is computed. In the experiments, we use the first *n*=5 values of the processed data. Several unreported experiments show that bigger *n* values do not affect the results significantly. Preprocessed input data for the proposed classifier system is shown in Figure 6.

Terrain classification dataset contains five inputs and four outputs. The dataset has 320 total samples. As in the previous experiment, this experiment is also repeated 30 times. For each run, 60% of the dataset is randomly divided into train and the rest is selected as test subsets. Average and standard deviation PCCS results of the 30 independent runs are given in Table 7.

As can be seen from Table 7, from best to worst, the algorithms can be ordered as ABC, GA, GD and KF, respectively. GA and ABC has smaller standard deviations then GD and KF which shows the robustness of population based intelligent optimization algorithms then traditional training algorithms. As a result, it can be said that ABC is better than the others from the point of view of higher average PCCS results and lower standard deviations.

**Figure 7.**
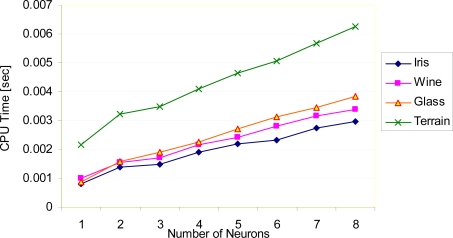
Evaluation CPU time of the RBF network.

Figure 7 shows the evaluation CPU time of the RBF network after training stage. As can be seen from the figure, number of neurons located in the hidden layer of the RBF network affects the CPU time of the RBF network in real-time applications. While the number of neurons increase, CPU time increase proportionally. In the experiments, an Intel Q6600 2.4 GHz personal computer and MATLAB software are used.

## 7. Conclusions

In this study, the Artificial Bee Colony (ABC) Algorithm, which is a new, simple and robust optimization algorithm, has been used to train radial basis function (RBF) neural networks for classification purposes. First, well-known classification problems obtained from UCI repository have been used for comparison. Then, an experimental setup has been designed for an inertial sensor based terrain classification. Training procedures involves selecting the optimal values of the parameters such as weights between the hidden layer and the output layer, spread parameters of the hidden layer base function, center vectors of the hidden layer and bias parameters of the neurons of the output layer. Additionally, number of neurons in the hidden layer is very important for complexity of the network structure. The performance of the proposed algorithm is compared with the traditional GD, and novel KF and GA methods. GD and KF methods are derivative based algorithms. Trapping a local minimum is a disadvantage for these algorithms. The GA and ABC algorithms are population based evolutional heuristic optimization algorithms. These algorithms show better performance than derivative based methods for well known classification problems such as Iris, Glass, and Wine and also for experimental inertial sensor based terrain classification. However, these algorithms have the disadvantage of a slow convergence rate. If the classification performances are compared, experimental results show that the performance of the ABC algorithm is better than those of the others. The success of the classification results of test problems are superior and also correlates with the results of many papers in the literature. In real-time applications, number of neurons may affect the time complexity of the system. For terrain classification problem, it is proved that inertial measurement units can be used to identify the terrain type of a mobile vehicle. The results of terrain classification problem are reasonable and may help to the planning algorithms of autonomous ground vehicles.

The main contributions of this study can be summarized as:

- Training algorithms of RBF networks significantly affect the performance of the classifier.
- Complexity of the RBF network is increased with the number of neurons in the hidden layer.
- GD and KF offer faster training but tolerable classification performance. GA and ABC show better performance than others.
- ABC is the applied for the first time to RBF training for classification problems in this study.
- ABC is more robust and requires less control parameters than other training algorithms.
- ABC reaches the best score of GD and KF using only two neurons, while GD and KF use eight neurons. Therefore, the complexity of the RBF-ABC scheme is much less than those of others in the real-time usage after training.
- Proposed RBF structure includes the training of spread parameters for each hidden neuron and bias parameters for the output layer which is also newly applied for RBF training.
- Terrain classification by using an inertial sensor and RBF network is achieved with 80% success rate.

## Figures and Tables

**Table 1.** Characteristics of the UCI dataset.

	**Inputs**	**Outputs**	**Total Samples**	**Training Samples**	**Test Samples**
**Iris**	4	3	150	90	60
**Wine**	13	3	178	106	72
**Glass**	9	2	214	128	86

**Table 2.** Control parameters of GA.

**Population size**	**Parameter count**
Number of generations	4,000
Selection type	Roulette
Mutation type	Uniform
Mutation rate	0.05
Crossover type	Single point
Crossover ratio	0.8

**Table 3.** Control parameters of ABC.

**Population Size**	**Parameter count**
Number of generations/cycles	4,000
Limit (ABC)	400

**Table 4.** Statistical PCCS results of Iris dataset.

		**Hidden Layer Neurons**							
		**1**	**2**	**3**	**4**	**5**	**6**	**7**	**8**
**GD**	Train	65,9 (2,7)	85,3 (6,6)	93,4 (3,4)	95,3 (3,4)	95,2 (2,7)	95,2 (7,2)	97,0 (3,0)	97,7 (1,3)
	*Test*	*60,4 (5,2)*	*82,3 (8,6)*	*90,7 (3,8)*	*91,6 (4,1)*	*94,5 (2,4)*	*91,8 (7,6)*	*93,8 (2,6)*	*93,9 (3,1)*
**KF**	Train	60,7(9,2)	66,4 (10,8)	81,6 (8,4)	84,5 (12,8)	88,3 (10,7)	91,6 (8,6)	94,2 (4,6)	95,6 (4,2)
	*Test*	*53,6(13,6)*	*63,3 (13,0)*	*77,9 (12,0)*	*83,8 (15,4)*	*85,5 (11,7)*	*88,8 (8,8)*	*91,2 (3,5)*	*91,8 (5,3)*
**GA**	Train	63,5 (12,3)	89,9 (8,6)	94,1 (3,8)	96,1 (2,0)	96,0 (1,7)	96,6 (1,9)	97,4 (1,4)	97,1 (1,1)
	*Test*	*58,9 (12,3)*	*88,1 (9,6)*	*91,9 (5,3)*	*94,3 (3,9)*	*95,5 (2,9)*	*94,6 (3,9)*	*95,9 (2,2)*	*96,1 (2,5)*
**ABC**	Train	**70,6 (5,2)**	**96,1 (1,5)**	**97,1 (1,6)**	**97,9 (1,3)**	**97,5 (1,6)**	**97,8 (1,3)**	**98,0 (1,3)**	**98,0 (1,0)**
	*Test*	***65,6 (7,1)***	***93,2 (2,5)***	***93,8 (2,8)***	***95,8 (2,9)***	***96,2 (2,4)***	***96,1 (2,2)***	***96,3 (2,5)***	***96,2 (2,6)***

**Table 5.** Statistical PCCS results of Wine dataset.

		**Hidden Layer Neurons**							
		**1**	**2**	**3**	**4**	**5**	**6**	**7**	**8**
**GD**	**Train**	54,5 (13,0)	74,5 (18,1)	83,6 (13,5)	89,9 (7,0)	92,1 (10,1)	95,1 (4,4)	97,0 (2,6)	97,1 (2,0)
	***Test***	*49,1 (16,0)*	*70,4 (19,2)*	*81,1 (13,1)*	*86,1 (11,7)*	*91,8 (10,8)*	*91,8 (6,8)*	*93,7 (5,5)*	*96,7 (2,6)*
**KF**	**Train**	52,9(8,4)	56,1 (14,6)	72,5 (17,9)	85,6 (14,5)	83,4 (15,1)	90,5 (13,3)	91,3 (15,4)	97,1 (3,0)
	***Test***	*47,2 (11,2)*	*52,5 (15,7)*	*68,8 (19,6)*	*79,6 (16,0)*	*80,9 (17,3)*	*85,6 (16,1)*	*88,9 (15,8)*	*94,4 (4,9)*
**GA**	**Train**	68,7 (7,8)	96,9 (2,8)	98,8 (1,0)	98,8 (1,3)	99,1 (1,0)	99,5 (0,8)	99,6 (0,6)	99,8 (0,5)
	***Test***	*65,7 (8,1)*	*93,8 (4,0)*	*97,5 (1,8)*	*96,7 (3,2)*	*97,2 (2,1)*	*97,2 (1,8)*	*97,3 (2,3)*	*97,2 (2,4)*
**ABC**	**Train**	**74,1 (5,3)**	**98,5 (1,5)**	**99,2 (0,8)**	**99,6 (0,6)**	**99,5 (0,5)**	**99,6 (0,5)**	**99,8 (0,4)**	**99,6 (0,5)**
	***Test***	***70,7 (8,5)***	***95,8 (2,9)***	***97,1 (1,7)***	***97,9 (1,9)***	***96,9 (2,1)***	***97,6 (1,7)***	***97,2 (2,0)***	***96,9 (2,2)***

**Table 6.** Statistical PCCS results of Glass dataset.

		**Hidden Layer Neurons**							
		**1**	**2**	**3**	**4**	**5**	**6**	**7**	**8**
**GD**	**Train**	84,8 (5,4)	88,3 (5,4)	90,6 (2,1)	90,8 (2,2)	92,8 (2,4)	93,0 (2,5)	91,7 (3,1)	91,8 (2,8)
	***Test***	*80,6 (7,1)*	*86,6 (5,8)*	*89,7 (3,5)*	*90,1 (4,0)*	*89,5 (3,4)*	*90,7 (2,4)*	*90,1 (4,3)*	*89,9 (4,5)*
**KF**	**Train**	76,6 (3,6)	84,8 (6,7)	89,6 (5,9)	91,0 (4,2)	92,0 (3,6)	94,1 (2,7)	93,8 (2,2)	94,7 (3,1)
	***Test***	*78,2 (5,6)*	*84,3 (7,4)*	*89,2 (4,3)*	*90,1 (3,0)*	*90,8 (4,5)*	*91,5 (3,3)*	*90,9 (2,8)*	*90,5 (5,6)*
**GA**	**Train**	82,6 (6,8)	92,6 (2,3)	94,0 (1,5)	94,7 (1,6)	95,4 (1,5)	96,2 (1,2)	95,9 (1,9)	96,5 (1,8)
	***Test***	*81,3 (8,1)*	*91,0 (2,3)*	*91,0 (2,8)*	*91,9 (2,4)*	*90,8 (2,6)*	*91,9 (2,5)*	*91,4 (3,8)*	*91,8 (2,9)*
**ABC**	**Train**	**92,2 (1,7)**	**94,1 (1,8)**	**95,5 (1,1)**	**95,3 (1,5)**	**96,3 (1,5)**	**96,7 (1,3)**	**97,0 (1,4)**	**96,9 (1,5)**
	***Test***	***88,8 (3,0)***	***91,7 (2,8)***	***91,2 (2,6)***	***92,6 (2,8)***	***91,9 (2,4)***	***91,8 (2,7)***	***91,7 (3,9)***	***92,1 (2,9)***

**Table 7.** Statistical PCCS results of Terrain Classification dataset.

		**Hidden Layer Neurons**							
		**1**	**2**	**3**	**4**	**5**	**6**	**7**	**8**
**GD**	**Train**	42,2 (6,1)	50,1 (6,6)	59,2 (8,3)	58,6 (7,3)	65,8 (5,0)	65,5 (5,8)	70,3 (5,2)	70,9 (6,0)
	***Test***	*36,6 (7,5)*	*46,9 (7,9)*	*55,2 (8,8)*	*55,8 (8,3)*	*60,7 (6,2)*	*62,0 (5,3)*	*66,9 (5,4)*	*66,2 (5,1)*
**KF**	**Train**	36,8 (7,1)	46,7 (6,9)	45,1 (11,8)	58,0 (10,0)	62,4 (12,3)	63,2 (11,7)	63,7 (11,7)	68,4 (9,4)
	***Test***	*33,8 (7,9)*	*42,3 (8,2)*	*41,2 (11,7)*	*53,5 (10,8)*	*59,4 (11,2)*	*60,9 (11,0)*	*59,7 (12,2)*	*65,0 (12)*
**GA**	**Train**	42,0 (5,5)	55,7 (6,9)	68,6 (4,3)	71,4 (3,2)	70,2 (3,5)	74,1 (3,1)	77,0 (3,3)	74,2 (2,7)
	***Test***	*37,4 (8,1)*	*48,3 (5,9)*	*65,1 (5,6)*	*64,8 (5,7)*	*66,7 (5,4)*	*69,5 (6,9)*	*73,2 (5,0)*	*72,1 (5,5)*
**ABC**	**Train**	**52,6 (4,3)**	**70,8 (4,2)**	**73,4 (2,6)**	**77,4 (3,2)**	**75,3 (1,6)**	**79,3 (1,2)**	**79,0 (1,9)**	**78,9 (2,6)**
	***Test***	***50,3 (3,2)***	***66,5 (2,5)***	***74,5 (5,0)***	***73,7 (2,0)***	***72,7 (3,0)***	***75,7 (4,1)***	***74,6 (1,3)***	***79,6 (3,0)***
